# Type-2 diabetic aldehyde dehydrogenase 2 mutant mice (ALDH 2*2) exhibiting heart failure with preserved ejection fraction phenotype can be determined by exercise stress echocardiography

**DOI:** 10.1371/journal.pone.0195796

**Published:** 2018-04-20

**Authors:** Guodong Pan, Srikar Munukutla, Ananya Kar, Joseph Gardinier, Rajarajan A. Thandavarayan, Suresh Selvaraj Palaniyandi

**Affiliations:** 1 Division of Hypertension and Vascular Research, Department of Internal Medicine, Henry Ford Health System, Detroit, MI United States of America; 2 Bone and Joint Center, Henry Ford Health System, Detroit, MI United States of America; 3 Department of Cardiovascular Sciences, Center for Cardiovascular Regeneration, Houston Methodist Research Institute, Houston, TX, United States of America; 4 Department of Physiology, Wayne State University, Detroit, MI, United States of America; University of Alabama at Birmingham, UNITED STATES

## Abstract

E487K point mutation of aldehyde dehydrogenase (ALDH) 2 (ALDH2*2) in East Asians intrinsically lowers ALDH2 activity. ALDH2*2 is associated with diabetic cardiomyopathy. Diabetic patients exhibit heart failure of preserved ejection fraction (HFpEF) i.e. while the systolic heart function is preserved in them, they may exhibit diastolic dysfunction, implying a jeopardized myocardial health. Currently, it is challenging to detect cardiac functional deterioration in diabetic mice. Stress echocardiography (echo) in the clinical set-up is a procedure used to measure cardiac reserve and impaired cardiac function in coronary artery diseases. Therefore, we hypothesized that high-fat diet fed type-2 diabetic ALDH2*2 mutant mice exhibit HFpEF which can be measured by cardiac echo stress test methodology. We induced type-2 diabetes in 12-week-old male C57BL/6 and ALDH2*2 mice through a high-fat diet. At the end of 4 months of DM induction, we measured the cardiac function in diabetic and control mice of C57BL/6 and ALDH2*2 genotypes by conscious echo. Subsequently, we imposed exercise stress by allowing the mice to run on the treadmill until exhaustion. Post-stress, we measured their cardiac function again. Only after treadmill running, but not at rest, we found a significant decrease in % fractional shortening and % ejection fraction in ALDH2*2 mice with diabetes compared to C57BL/6 diabetic mice as well as non-diabetic (control) ALDH2*2 mice. The diabetic ALDH2*2 mice also exhibited poor maximal running speed and distance. Our data suggest that high-fat fed diabetic ALDH2*2 mice exhibit HFpEF and treadmill exercise stress echo test is able to determine this HFpEF in the diabetic ALDH2*2 mice.

## Introduction

The prevalence of diabetes mellitus is increasing globally, particularly in Asia which constitutes almost 60% of the global diabetic patients [1]. The Diabetes Atlas published in 2013 reported that 366 million individuals are affected by diabetes, and 36% of those affected live in the Western Pacific region, with a significant proportion in East Asia [2]. Among the East Asians, around 700 million of them have E487K point mutation of ALDH2 (ALDH2*2)[3]. ALDH2*2 is associated with maternal inheritance of type-2 diabetes [4] and diabetic complications [5] including diabetic cardiac dysfunction [6].

In general, complications from cardiovascular disease is the leading cause of death in patients with diabetes [7, 8]. Additionally, the development of heart failure is more severe in patients with diabetes compared to non-diabetic patients[9]. This is due to impediments in early diagnosis and prevention [10, 11]. For instance, in patients with diabetes there is a lack of specific diagnostic criteria used to evaluate the onset of deterioration in cardiac function. In most cases, when the diabetic patient presents systolic dysfunction, it is generally too late to effectively recover heart function. In addition to diabetes being an independent risk factor for cardiovascular diseases [12], the absence of symptoms related to coronary heart disease (e.g., angina) often makes it a difficult disorder to diagnose and treat [13, 14]. Furthermore, evaluating abnormalities in left ventricular function in patients with type-2 diabetes is difficult because these patients typically exhibit a normal or preserved ejection fraction, and abnormalities in diastolic function are difficult to detect using regular echocardiography. These same problems extend to animal models of type-2 diabetes mellitus (T2DM), metabolic syndrome and other cardio-metabolic syndromes [15–17]. Therefore, monitoring deterioration in cardiac function over time after the onset of T2DM is difficult in mice. Additionally, in the clinical setting, self-reported heart failure-related symptoms such as dyspnea at rest or with exertion, early onset fatigue, exercise intolerance and palpitations all represent important disease defining information that cannot be self-reported or captured in mice.

Pertaining to this study, several epidemiological studies indicated that ALDH2 is critical in cardiovascular disease: inactive ALDH2 genotype is associated with a higher incidence of myocardial infarction [18, 19], angina [20] and hypertension [21] in humans. The cardioprotective effect of ALDH2 has been demonstrated using *in vivo* and *ex vivo* models of myocardial ischemia-reperfusion injury [22, 23]. ALDH2 is particularly important in the detoxification of aldehydes like 4HNE that accumulate under oxidative stress in the diabetic heart [23, 24]. ALDH2*2 is associated with increased oxidative stress in diabetes and can be linked to diabetic cardiomyopathy.

In the clinic, exercise stress echocardiography is performed in order to evaluate whether the myocardium is receiving adequate blood flow and oxygen under increased myocardial demand [25, 26]. This is also a methodology used to determine cardiac function in HFpEF [27] besides used for myocardial ischemic diseases [28]. In general, HFpEF which implicates preserved systolic function while showing sign of diastolic dysfunction is difficult to diagnose at early stage in patients and much harder in mice. Early diagnosis helps strategize for the proper treatment to prevent overt heart failure. Therefore, our goal is to develop a method to detect HFpEF in mice. Based on prior work in our laboratory, we found that ALDH2*2 mice develop cardiac dysfunction around 6 months after induction of T2DM by high-fat diet feeding in ALDH2*2 mice (unpublished). Therefore we decided to establish the stress echo method in ALDH2*2 mice with T2DM just before cardiac dysfunction develops (i.e., at 4 months).

In the current study, after performing echocardiography in conscious C57BL/6 and ALDH2*2 mice, as we have done earlier in mice [29, 30], we used the Bruce protocol (with modifications) to make the C57BL/6 and ALDH2*2 mice run on the treadmill until exhaustion and subsequently perform echocardiography.

The objectives of this study are to determine whether ALDH2*2 mice with type-2 diabetes exhibit the HFpEF phenotype and to evaluate the capability of our exercise echo stress test methodology in detecting this cardiac functional anomaly.

## Methods

### Induction of type-2 diabetes mellitus

12-week-old C57BL/6 and ALDH2*2 male mice with C57BL/6 background were fed a high-fat diet (60% of calories from fat, D12492, Research Diets) (Diabetic, DM) or normal chow (Control, Ctrl) until sacrifice (roughly 18 weeks) N = 9. After a month, mice with sustained elevated fasting blood glucose levels (>200 mg/dl) were regarded as diabetic mice and selected for further studies. Blood was collected from the tail vein and glucose was then measured with a glucometer. The body weights were also measured and recorded. The animal protocol was approved by the Henry Ford Health System Institutional Animal Care and Use Committee. It adheres to the guiding principles of the care and use of experimental animals in accordance with NIH guidelines. Henry Ford Hospital operates an AAALAC certified animal facility.

ALDH2*2 mice were inbred in-house after obtaining them as a gift from Dr. Daria Mochly-Rosen Lab in Stanford University. They were genotyped by Transnetyx Inc.

### Intraperitoneal glucose tolerance test (IPGTT)

The IPGTT was performed in mice from control and diabetic groups as explained elsewhere [29]. After fasting for 6 hrs, the mice were injected with 2g/kg D-glucose. Then, blood glucose levels were measured at 0, 30, 60, 90 and 120 min after D-glucose injection, using a glucometer.

### Measurement of serum insulin levels

As we described earlier [29], blood samples were spun and the separated serum was used to measure insulin levels using an ELISA kit (Crystal Chem Inc) as per the manufacturer’s instructions. Each value represents duplicate measurements of each sample.

### Cardiac function assessment by echocardiography in conscious mice

Left ventricular dimension and function were assessed in conscious mice to avoid the effects of anesthesia. We used an echocardiograph equipped with a 15-MHz linear transducer (Acuson c256) as described previously [29] [30]. We picked up the mice by their nape and held them firmly in the palm of one hand in the supine position, while the tail was held tightly between the last two fingers. The left hemithorax was carefully shaved, and a prewarmed ultrasound transmission gel (Parker Laboratory, Orange, NJ) was applied to the precordium. Transthoracic echocardiography was performed using an Acuson 256 equipped with a 15-MHz linear transducer (15L8) in a phased-array format, which offers 0.35-mm lateral resolution and 0.25-mm axial resolution, real-time digital acquisition, storage, and review capabilities. Generally, the heart was first imaged in the two-dimensional (2-D) mode of the parasternal long-axis view. From this view, an M-mode cursor was positioned perpendicularly to the interventricular septum and posterior wall of the LV, at the level of the papillary muscles. M-mode images were then obtained for measurement of wall thickness and chamber dimensions. The cursor was moved to the aortic root and the aortic dimension was obtained, after which a 2-D short-axis view of the mid-LV was produced at the chordal level by rotating the transducer clockwise 30–45°. Images from this view were used to measure LV cross-sectional area. Aortic flow velocity was measured with pulsed-wave Doppler on the parasternal long-axis view. The sample volume cursor was placed in the aortic root and the transducer angled slightly, which allowed aortic flow parallel to the interrogation beam so that maximum aortic flow velocity was obtained easily. Images were stored in digital format on a magnetic optical disk for review and analysis.

### Calculation of cardiac relaxation rate

Cardiac relaxation rate (CRR), an index of cardiac diastolic function, was calculated by measuring the difference between diastolic and systolic diameter {in centimeter (CM)} over a given period of time {(in seconds (S)}. CRR is therefore the slope of the diastolic and systolic curves in the M-mode image in echocardiography. The time was calculated using the heart rate recordings. CRR was calculated automatically by the software and provided as slope. However, this slope can be calculated manually as well by using the following formula:

CRR (cm∕s)=LV dimension during diastole−LV dimension during systole∕time

### Acute progressive maximal exercise test (exhaustion test)

The control and diabetic ALDH2*2 mice were brought to the treadmill room for 2 hours in advance so that they could acclimate to the environment. Furthermore, they were allowed to explore and acclimatize to the treadmill for at least 3 minutes. Next, the mice were subjected to the exhaustion test used in previous studies [16, 31]. Specifically, mice were placed on the treadmill (0° incline for entire experiment) and then subjected to the activation of the shock grid. The treadmill speeds were then increased until exhaustion occurred: (speed, duration)—(0 m/min, 5 min), (6 m/min, 5 min), (7, 8, 9, and 10 m/min, 30s each), (11m/min, 1 min), (12, 13, 14, and 15 m/min, 2 min each), and (+1 m/min, each 1 min thereafter). Exhaustion (endpoint for treadmill cessation) was defined as the point at which mice maintained continuous contact with the shock grid for 5 seconds.

### Post-exercise echocardiography

As soon as the mice finished stress treatment, we performed conscious echocardiography on them to record their functional changes as described above.

### Determination of mouse brain natriuretic peptide (BNP) levels in the serum

Blood samples were spun and the separated serum was used to measure BNP levels using an ELISA kit (MyBioSource.com) as per the manufacturer’s instructions. Each value represents duplicate measurements of each sample.

### Measurement of cardiomyocyte hypertrophy

Myocardial sections were stained with hematoxylin-eosin to measure cardiomyocyte hypertrophy by quantifying the myocyte cross-sectional area using Adobe Photoshop (Adobe Systems Inc. San Jose, CA) and MicroSuite (Olympus America Inc. Central Valley, PA) software. Relatively circular cardiomyocytes with the nucleus in the center were included for quantification of each high power field. We scored at least 100 cardiomyocytes for each sample.

### Statistical analysis

Data are presented as mean ± standard error of the mean (SEM). We used One-way ANOVA for group comparisons. The difference between control and diabetic groups were analyzed by using the unpaired Student t-test. The difference between the time points from before and after exercise stress testing was analyzed by paired Student t- test.

## Results

### High-fat feeding induces type-2 diabetes in C57BL/6 and ALDH2*2 mice

**Fig 1:**
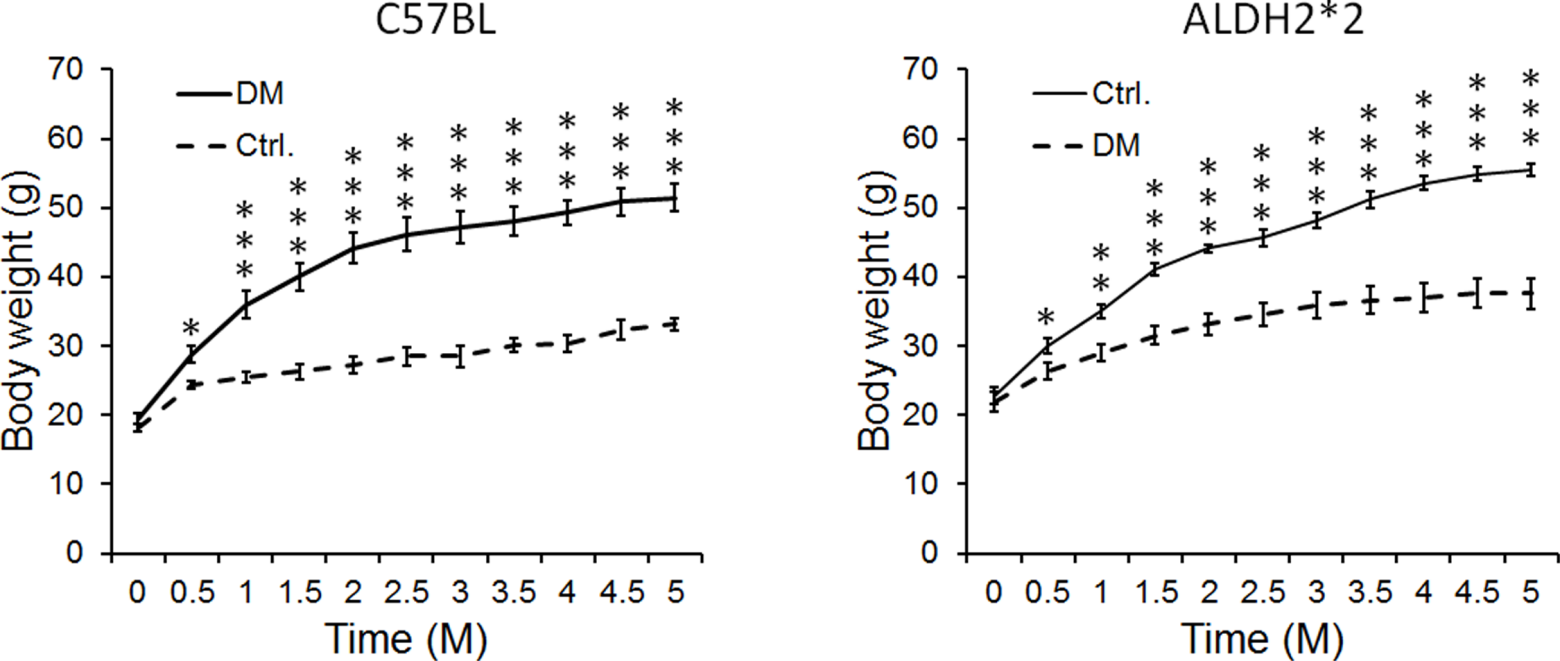
High-fat diet induces obesity in C57BL and ALDH2*2 mutant mice. Body weight increase in high-fat fed C57BL and ALDH2*2 mutant diabetic mice (DM) compared to their respective non-diabetic controls (Ctrl.). Data are presented as mean ± standard error of the mean (SEM). ^*^p<0.05, ^**^p<0.01 and ^***^p<0.001 vs Respective Ctrl.

**Fig 2:**
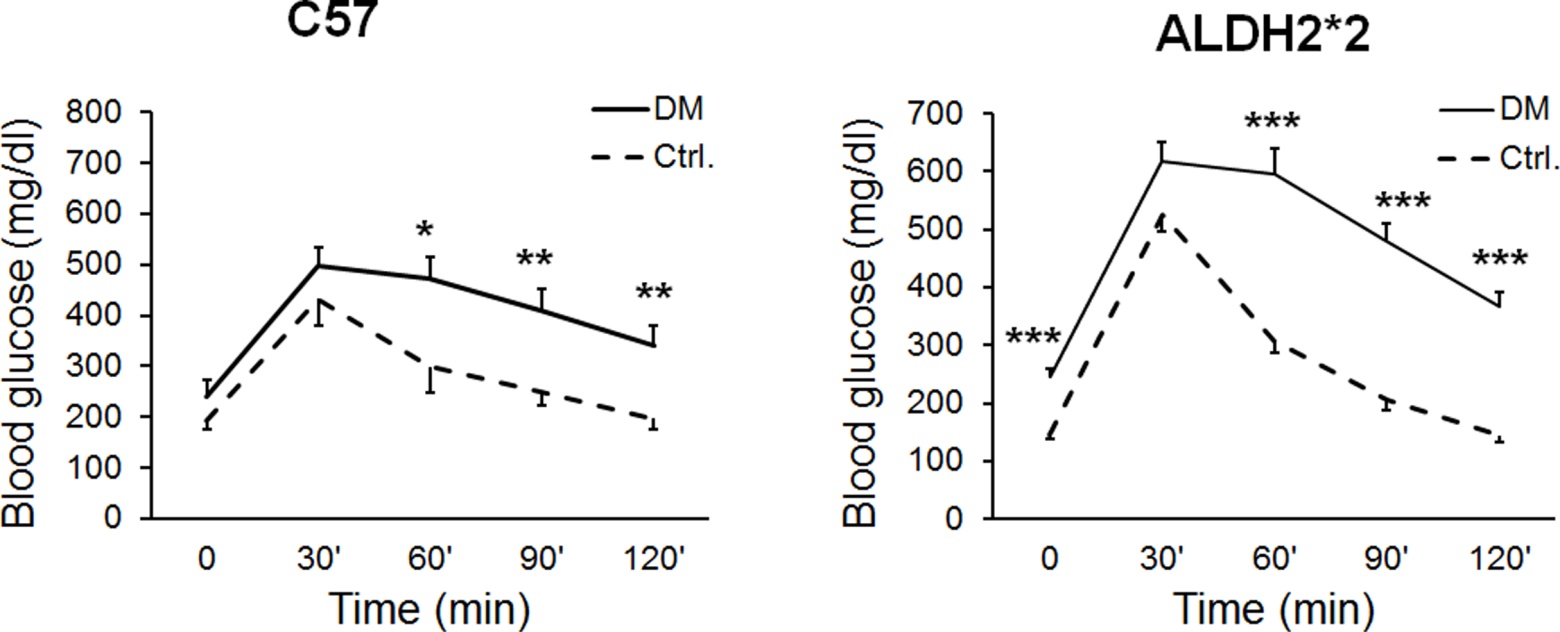
High-fat diet induces hyperglycemia and glucose intolerance in C57BL and ALDH2*2 mutant mice. Increase in blood glucose levels (refer values at the zero minutes) and glucose intolerance with IPGTT up to 120 minutes was observed in high-fat fed C57BL and ALDH2*2 mutant diabetic mice (DM) compared to their respective non-diabetic controls (Ctrl.).Data are presented as mean ± standard error of the mean (SEM). ^*^p<0.05, ^**^p<0.01 and ^***^p<0.003 vs Respective Ctrl.

**Fig 3:**
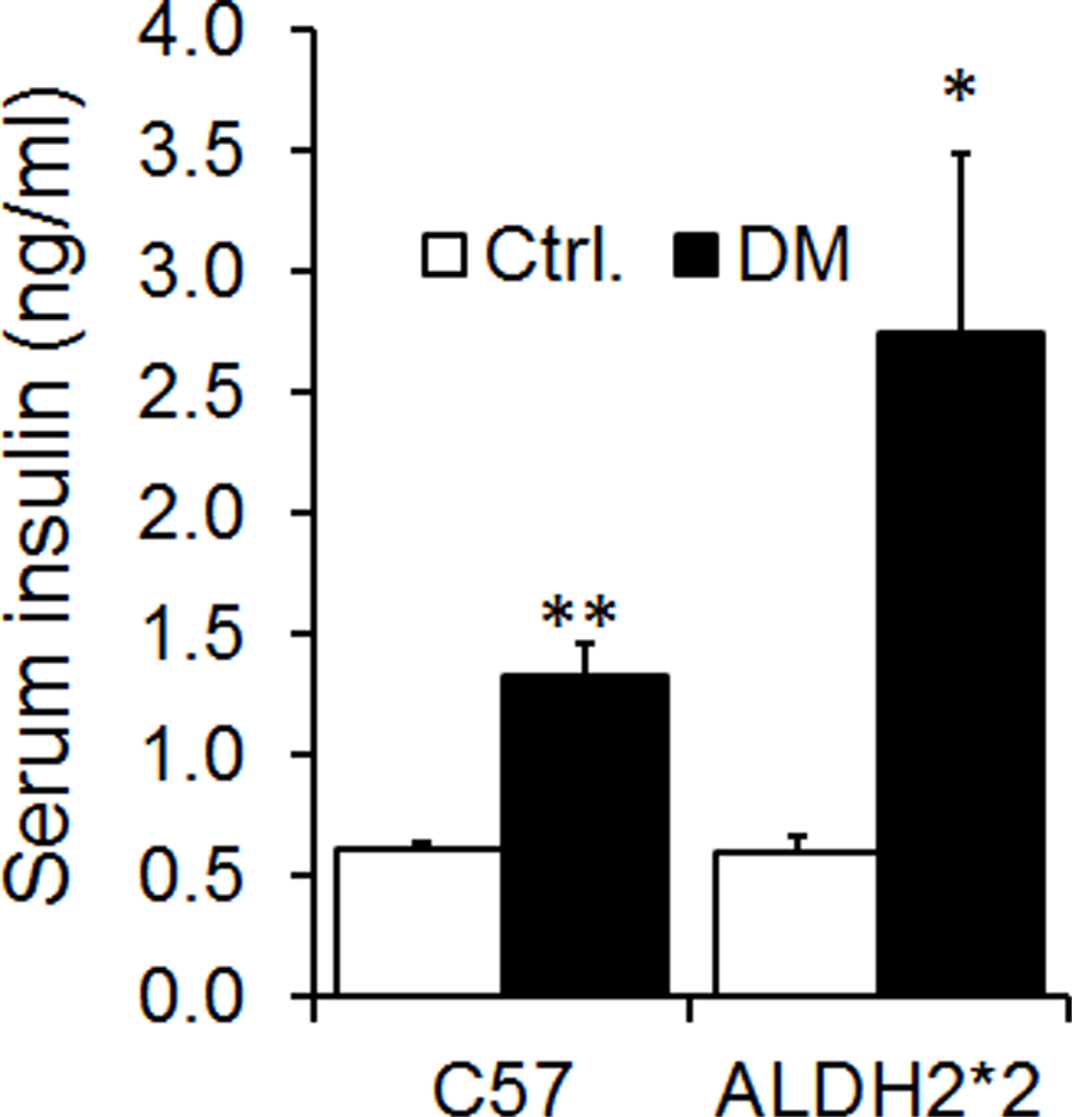
High-fat diet induces hyperinsulinemia in C57BL and ALDH2*2 mutant mice. Serum insulin levels were measured using insulin ELISA kit. ^*^p<0.05 and ^**^p<0.01 vs. Respective Ctrl.

High-fat diet feeding to C57BL/6 and ALDH2*2 mice resulted in increased body weight (obesity) (Fig 1). High-fat diet also increased blood glucose levels at basal (hyperglycemia) and upon glucose challenge, the mice exhibited glucose intolerance (insulin resistance) when compared to normal chow-fed C57BL/6 and ALDH2*2 mice (Fig 2). The high-fat diet also increased serum insulin levels (hyperinsulinemia) in those mice (Fig 3). This indicates that high-fat fed C57BL/6 and ALDH2*2 mice exhibit type-2 diabetes.

### Impaired hemodynamic changes in diabetic ALDH2*2 mice after exercise stress

**Fig 4:**
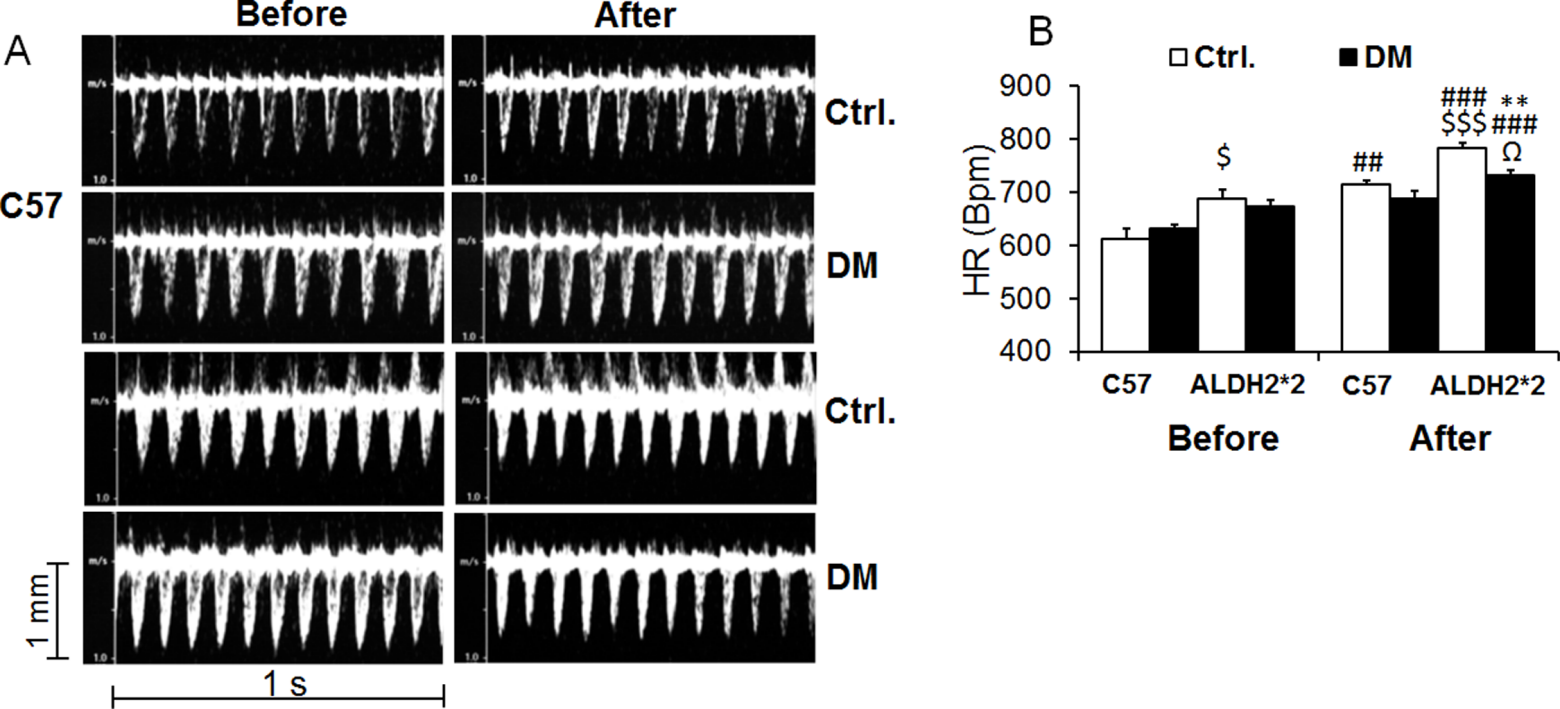
Increase in heart rate with exercise stress. Increase in heart rate is lower in diabetic mice compared to control mice. Data are presented as mean ± standard error of the mean (SEM). ^$^p<0.05 and ^$ $ $^p<0.001 vs C57 Ctrl. ^**^p<0.01 vs. Respective Ctrl. ^##^p<0.01 and ^###^p<0.001 vs before exercise stress of same group; ^Ω^p<0.05 vs. C57DM.

After 4 months, we found a significant increases in the heart rate after exercise in both C57BL/6 and ALDH2*2 mice free of diabetes and ALDH2*2 mice with 4 months of type-2 diabetes (Fig 4A and 4B).

**Fig 5:**
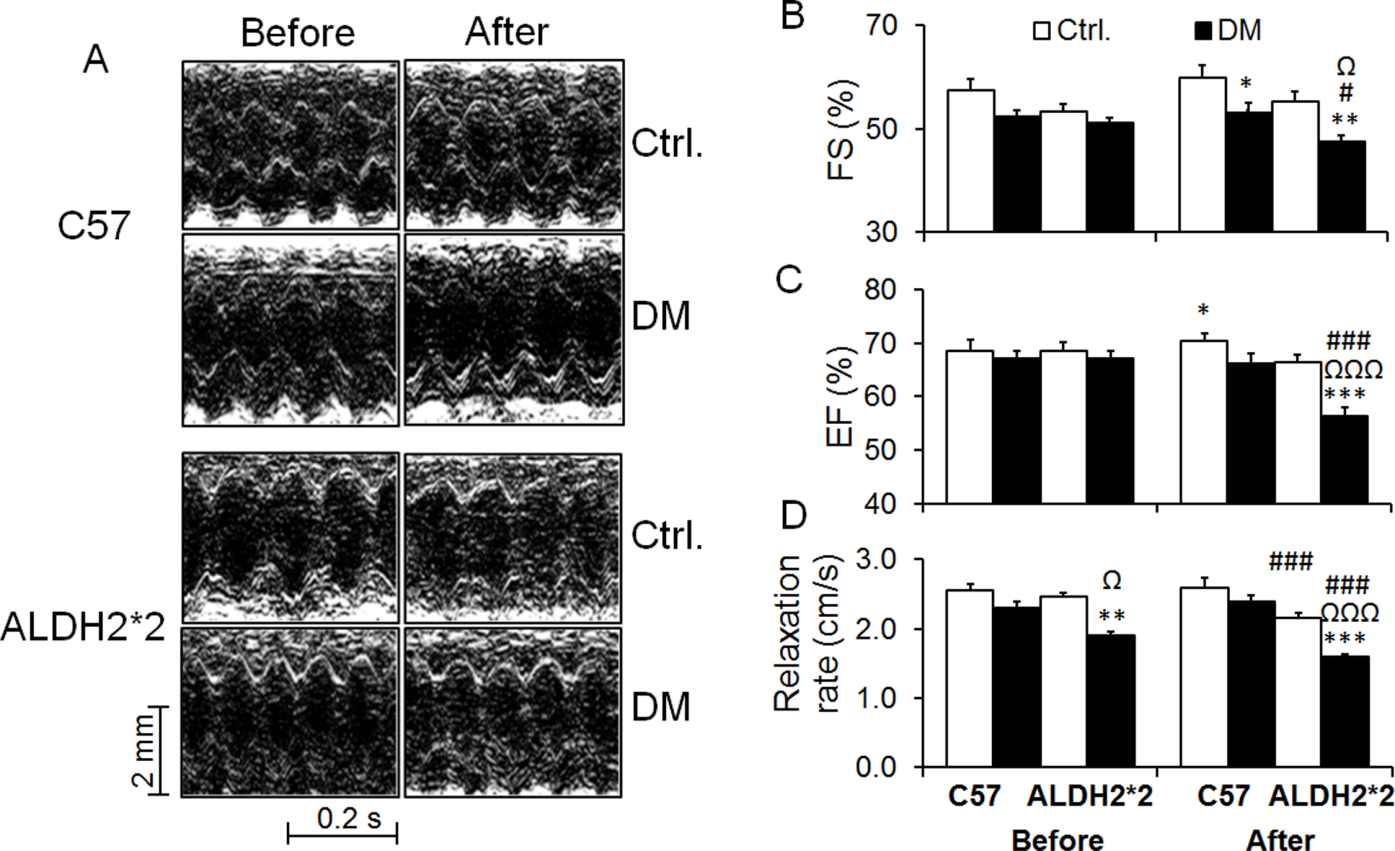
Changes in systolic contractile functional parameters with exercise stress. Larger decrease in %FS (B) and %EF (C) in diabetic mice compared to control mice. Decrease in diastolic functional parameter, cardiac relaxation rate (CRR) (D) is observed in diabetic mice compared to control mice. Data are presented as mean ± standard error of the mean (SEM). ^*^p<0.05, ^**^p<0.01 and ^***^p<0.001 vs. Respective Ctrl.; ^#^p<0.05 and ^###^p<0.001 vs before exercise stress of same group; ^Ω^p<0.05 and ^ΩΩΩ^p<0.001 vs. C57DM.

% fractional shortening (FS) (Fig 5A and 5B) and % ejection fraction (EF) (Fig 5A and 5C) were significantly lower in diabetic ALDH2*2 mice after exercise when compared to control ALDH2*2 mice as well as C57BL/6 diabetic mice. Notably, there was no decrease in % FS and % EF in diabetic C57BL/6 and diabetic ALDH2*2 mice relative to control C57BL/6 and ALDH2*2 mice before exercise (at rest) (Fig 5A, 5B and 5C). CRR, a diastolic dysfunction index, was significantly decreased before and after exercise stress in diabetic C57BL/6 and diabetic ALDH2*2 mice (Fig 5A and 5D).

### Diabetic ALDH2*2 mice run shorter time and distance

**Fig 6:**
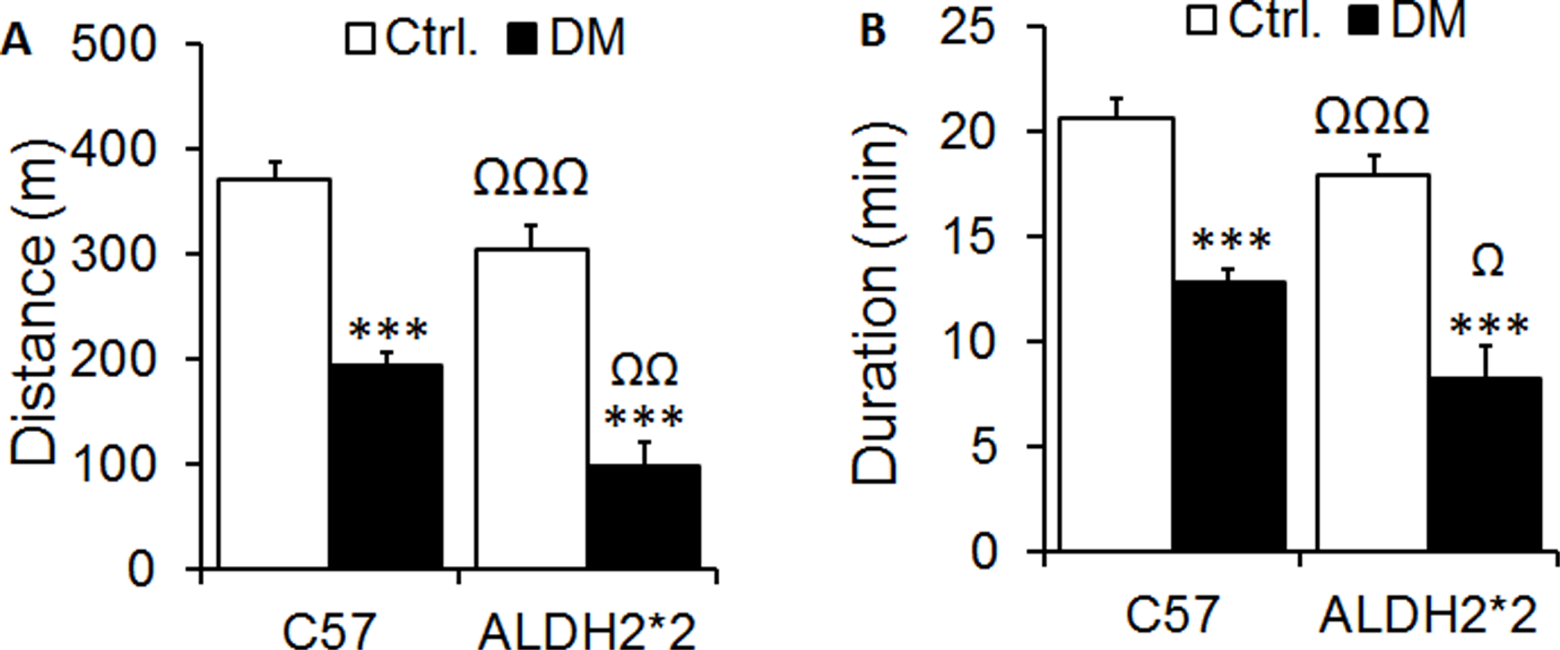
Running distance and duration of control and diabetic mice. Lower running duration (A) and distance (B) in diabetic mice compared to control mice. Data are presented as mean ± standard error of the mean (SEM). ***p<0.001 vs. C57 Ctrl.; Ωp<0.05 and ΩΩΩp<0.001 vs C57 DM.

We found that type-2 diabetic C57BL/6 and ALDH2*2 mice (at 4 months after diabetes induction) run for shorter distance (Fig 6A) and duration (Fig 6B) until exhaustion, compared to control C57BL/6 and ALDH2*2 mice. The ALDH2*2 diabetic mice ran for a significantly shorter duration and distance (Fig 6A and 6B) than C57BL/6 diabetic mice. In summary, the running indices indicate lower exercise tolerance in diabetic ALDH2*2 mice compared to their non-diabetic controls and C57BL/6 diabetic mice.

### A positive correlation between running distance and cardiac function

**Fig 7:**
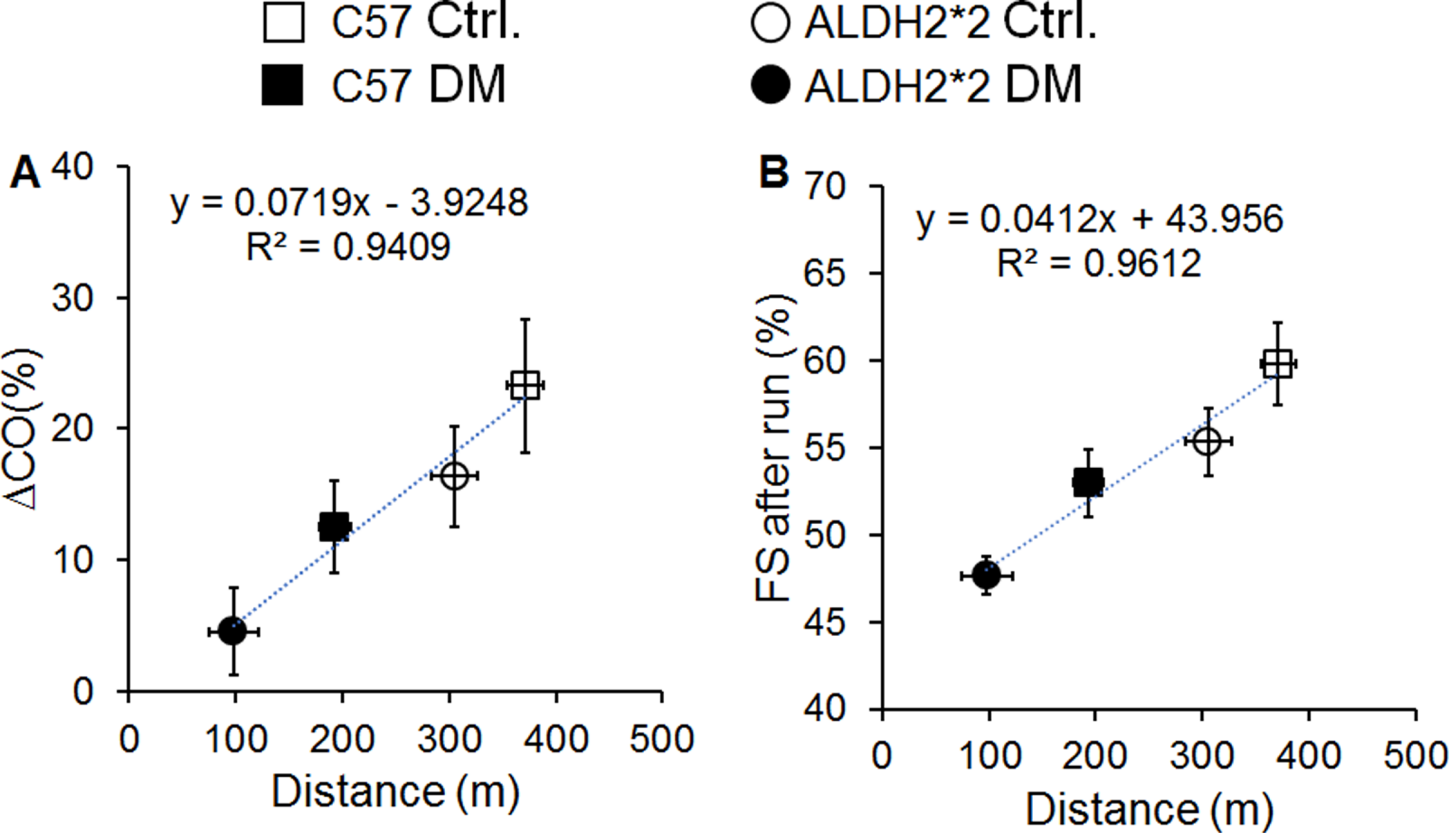
Correlation graphs. A positive correlations between % change in cardiac output (CO) and running distance (A) as well as % FS after run and running distance (B).

Ultimately, we found positive correlations between % change in cardiac output (∆CO) and running distance (Fig 7A) as well as % FS after run and running distance (Fig 7B). As depicted in the figure the diabetic ALDH2*2 mice have lower % ΔCO and % FS compared to running distance.

### Increased hypertrophy in diabetic ALDH2*2 mice

**Fig 8:**
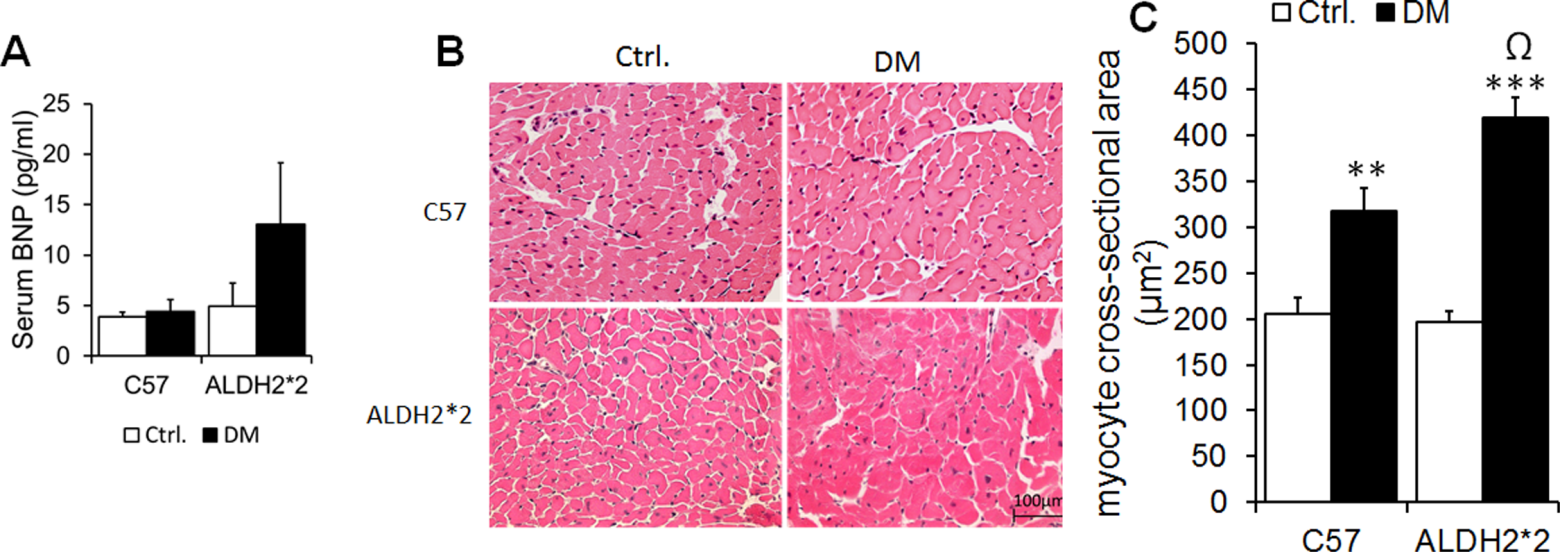
Cardiomyocyte hypertrophy in diabetic mice. Serum BNP levels (A). Cardiomyocyte hypertrophy (B) and its quantification data (C) Data are presented as mean ± standard error of the mean (SEM). ^**^p<0.01 and ***p<0.001 vs. Respective Ctrl.; Ωp<0.05 vs C57 DM.

We found slightly increased serum BNP levels (Fig 8A) and significantly increased cardiomyocyte hypertrophy as depicted by increased cross-sectional area (Fig 8B and 8C) in diabetic ALDH2*2 mice compared to their non-diabetic controls and C57BL/6 diabetic mice.

## Discussion

In ALDH2*2 mice type-2 diabetic mice, we showed numerous abnormalities in exercise induced cardiac function, including chronotropic incompetence (smaller increases in heart rate), cardiac output, decrease in contractile function as measured by both reduced % FS and % EF, and poor diastolic function as measured by CRR.

In the current study, we induced type-2 diabetes in ALDH2*2 mice using high-fat diet. In our hands, it takes around 6 months to develop cardiac dysfunction after T2DM induction in the ALDH2*2 mutant mice. Even after 4 months, our type-2 diabetic ALDH2*2 mice exhibited HFpEF phenotype at rest: the cardiac contractile functional parameters %FS and %EF did not decrease in diabetic mice at rest (comparable to control mice). However, immediately after exercise stress, we observed significant decreases in % FS and % EF in ALDH2*2 diabetic mice compared to both ALDH2*2 control mice and most importantly, C57BL/6 diabetic mice. The diastolic functional parameter CRR was lower in ALDH2*2 diabetic mice compared to systolic function, both before and after exercise stress.

Based on the presentation of cardiac dysfunction, heart failure is divided into HFrEF and HFpEF[32]. HFrEF is related to systolic dysfunction and characterized by the inability of the myocardium to contract and eject enough blood. It is easily observable. On the other hand, HFpEF may exhibit diastolic dysfunction while systolic function is preserved. It has been reported that HFpEF constitutes more than 50% of elderly HF patients [33] exhibiting reduced exercise tolerance. Thus, the significance of our current study is huge.

In the current study, we found ALDH2*2 diabetic mice exhibit diastolic dysfunction and preserved systolic function at rest, similar to clinical HFpEF. Moreover, HFpEF patients display exercise intolerance [34]. Cardiac response to exercise is a critical functional measure and an index of quality of life [35]. Due to these reasons, stress echo testing plays an important role in the diagnosis of HFpEF in diabetic [36] and non-diabetic patients [35]. Since our diabetic animals exhibit HFpEF, we subjected them to exercise stress echo test.

Exercise performance, such as maximal running speed and distance, are closely related to maximal oxygen consumption (VO_2_ max) [37]. The relationship between maximal cardiac output and VO_2_ max during exercise has generally been assumed to be linear in human subjects. In this study, type 2 diabetic ALDH2*2 mice exhibited remarkably poor exercise capacities, including decreased maximal running speed, and shortened distance and duration, which point to decreased VO2 max and impaired cardiac function. But there could be other significant contributions from changes in skeletal muscle function, nervous system effects, vascular component and overall energy metabolism especially when the heart doesn’t respond adequately. For this reason, we performed echocardiography both before and immediately after exercise stress to measure the changes of cardiac function parameters.

We found that the cardiac functional reserve was lower in diabetic ALDH2*2 mice compared to control ALDH2*2 mice. There was an increase in heart rate and cardiac output after exercise stress in both control and diabetic ALDH2*2 mice. However, the increase in these parameters immediately after exercise was relatively lower in diabetic ALDH2*2 mice compared to control ALDH2*2 mice. The core of exercise response was an increase in cardiac output. Mostly, this increase was due to excessive exertion of the heart due to exercise. Thus, our data suggest that type-2 diabetic ALDH2*2 mice have a weaker heart compared to normal chow fed control ALDH2*2 mice.

A cardiac-centric functional capacity assessment in response to exercise test is difficult to analyze due to the presence of aforementioned extra-cardiac factors. However, using conscious echocardiography to measure the heart function is advantageous for this test. General anesthesia suppresses cardiac function [38] via either a direct or indirect action on the central nervous system and heart. The use of anesthesia may further jeopardize the functional assessments in HFpEF. Therefore, we utilize conscious echocardiography, which was established two decades ago in our laboratories [30] and now adapted successfully for diabetic models too [29].

Furthermore, decrease in cardiac systolic functional parameters % FS and % EF only in diabetic ALDH2*2 mice but not with C57BL/6 diabetic mice with conscious echocardiography were apparent only after acute exercise stress, not before (existence of the diastolic dysfunction as measured by CRR before exercise). This implicates the HFpEF phenotype in our animal model. In our experiments, the apparent cardiac functional deterioration in diabetic animals occur around 6 months. Therefore, it is crucial to establish a method that determines cardiac dysfunction at an earlier stage. We believe that this is the first time that cardio echo test has been conducted in mice and particularly in diabetic mice. We specifically chose 4 months post-DM induction as our time point for exercise test as it is closer to 6 months (which is when the actual cardiac dysfunction started to present itself even at rest). A time course study will identify the earliest time period at which echo stress test will show cardiac dysfunction in type-2 diabetic models.

So far the best method in measuring cardiac reserve function in mice was employing treadmill exercise in real time with a telemeter [39]. In the telemetry study, the transmitter body of the telemetry device was placed subcutaneously on the left flank of the mouse. The pressure sensor, located within the tip of a catheter, was inserted into the left ventricle through an apical stab wound in order to produce continuous, non-tethered recordings of pulsatile LV pressure [39]. In comparison to our technique, this technique has merits and drawbacks. The merits are: measurement of cardiac function is continuous during exercise in conscious mice; no restraining of mice is required. In this method, a 2-fold increase in cardiac output with exercise was observed depicting the cardiac reserve. The disadvantages are: fine surgical skills are necessary to avoid post-surgical morbidity, mortality as well as noise; telemetry probes and catheters, which are expensive, would require delicate handling, periodic replacement and refurbishing. Furthermore, periodic functional measurements in long-term models such as ours require an extended presence of the probes and catheters *in situ* which may have its own difficulties and complications. Alternatively, repeated surgeries to place the probes and catheters at different time-points may bring post-surgical complications.

Apart from exercise stress test, β adrenergic receptor agonists such as dobutamine and isoproterenol was administered to challenge the heart so that cardiac reserve capacity could be determined [40, 41]. However, this method is not suitable to be employed in heart failure or diabetic animal models like ours since the diabetic hearts have decreased β adrenergic receptors [42] and signaling [41]. Our diabetic mice would require higher doses of β adrenergic receptor agonists to elicit a response due to lower density of cardiac β receptors which could be detrimental to control animals with normal cardiac β adrenergic receptor levels and signaling. In fact, in our earlier attempt, the control animals died abruptly with the dose of isoproterenol used, but not the diabetic animals (unpublished observation). Therefore, we strongly believe our methodology of cardiac echo stress test in mice may be useful for measuring cardiac functional reserve, exercise capacity and HFpEF.

Another surrogate marker often used in these procedures is exercise capacity [15]. There was a significant decrease in both running distance and duration in diabetic ALDH2*2 mice compared to control ALDH2*2 mice as well as C57BL/6 diabetic mice. The short running distance/time may be partially attributed to functional, metabolic, biochemical and structural abnormalities in the diabetic mice at the skeletal muscle, respiratory, nervous and cardiovascular systems. However, there were very positive correlations between running distance and cardiac reserve capacity as well as running distance and systolic function (%EF). Our mice were untrained; however, they were allowed to acclimatize to the treadmill prior to the experiment. The running protocol we employed was a means to exhaust the mice so that echocardiography could be performed to measure cardiac dysfunction. Nevertheless, it has been shown in previous studies that diabetic animals have poor running capacity [43]. The current study was not designed to understand the mechanisms associated with this poor running; rather, it establishes a feasible methodological development for cardiac stress echo.

For the first time, our study indicates type-2 diabetic ALDH2*2 mice exhibit HFpEF. We also found that our mice also present an increase in cardiac hypertrophy in ALDH2*2 diabetic mice. Our study may trigger some research in ALDH2*2 diabetic patients in East Asia as well as the immigrants from those countries living in the Western world. Additionally, we could show a better method to determine this specific and very difficult pathological condition. The ease of developing transgenic models, recapitulation of human genetic conditions, diseases and therapeutic interventions makes the mice attractive model systems for studying cardiovascular diseases. Therefore, our attempt to develop a cardiac echo stress test in mice will be useful in understanding the pathophysiology, prognosis and drug screening of heart failure models. The design of this methodology itself validates the HFpEF phenotype of a given animal model and also offers the means to measure cardiac systolic dysfunction in HFpEF. Further studies on various type-2 diabetic mice such as db/db and ob/ob mice and different mouse strains are required to consolidate our findings and expand them to various drug discovery research projects.

In summary, cardiac response to exercise is both a critical functional measure and an index of quality of life in clinic. Our methodology of measuring cardiac function by echocardiography just before and after exercise stress in type-2 diabetic mice is unique and novel. Since it also mimics the clinical scenario, we expect that our method will aid translational discoveries.
