# Correction: Type-2 diabetic aldehyde dehydrogenase 2 mutant mice (ALDH 2*2) exhibiting heart failure with preserved ejection fraction phenotype can be determined by exercise stress echocardiography

**DOI:** 10.1371/journal.pone.0203581

**Published:** 2018-08-30

**Authors:** Guodong Pan, Srikar Munukutla, Ananya Kar, Joseph Gardinier, Rajarajan A. Thandavarayan, Suresh Selvaraj Palaniyandi

In Fig 1, the legend in the ALDH2*2 graph is incorrect. The solid line should indicate “DM” and the broken line should indicate “Ctrl.”. Please see the correct Fig 1 here.

**Fig 1:**
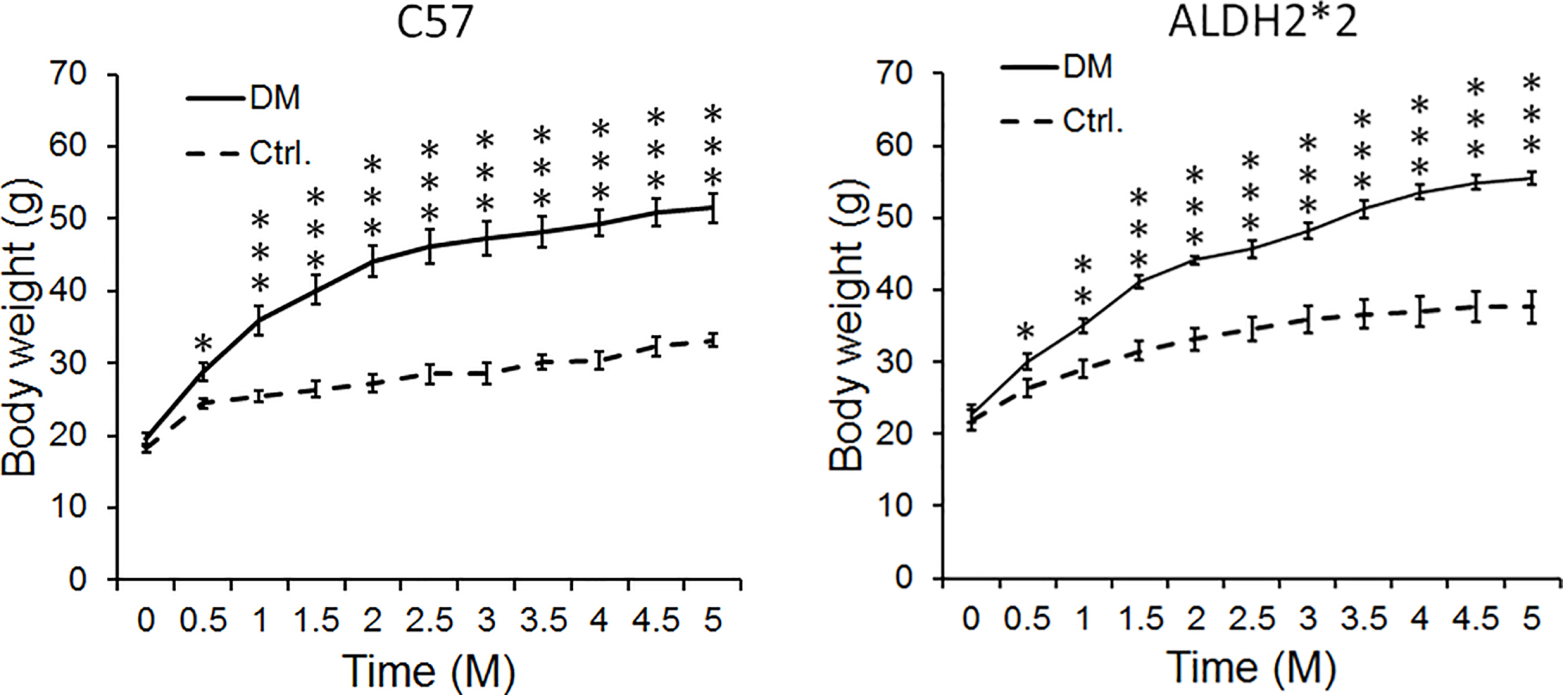
High-fat diet induces obesity in C57BL and ALDH2*2 mutant mice. Body weight increase in high-fat fed C57BL and ALDH2*2 mutant diabetic mice (DM) compared to their respective non-diabetic controls (Ctrl.). Data are presented as mean ± standard error of the mean (SEM). *p<0.05, **p<0.01 and ***p<0.001 vs Respective Ctrl.
